# Medicinal plants as therapeutic options for topical treatment in canine dermatology? A systematic review

**DOI:** 10.1186/s12917-019-1854-4

**Published:** 2019-05-27

**Authors:** Milena Tresch, Meike Mevissen, Hannah Ayrle, Matthias Melzig, Petra Roosje, Michael Walkenhorst

**Affiliations:** 1 Division Veterinary Pharmacology & Toxicology, Department Clinical Research and Veterinary Public Health, Vetsuisse Faculty, University of Bern, Laenggassstrasse 124, 3012 Bern, Switzerland; 2 Department of Livestock Sciences, Research Institute of Organic Agriculture (FiBL), Ackerstrasse 113, Postbox 219, 5070 Frick, Switzerland; 3 Dahlem Centre of Plant Sciences, Institute of Pharmacy, Freie Universität Berlin, Koenigin-Luise-Strasse 2+4, 14195 Berlin, Germany; 4 Division of Clinical Dermatology, Department of Clinical Veterinary Medicine, Vetsuisse Faculty, University of Bern, Laenggassstrasse 124, 3012 Bern, Switzerland

**Keywords:** Dog, Dermatology, *Calendula officinalis* L., *Hypericum perforatum* L., *Matricaria chamomilla* L., *Salvia officinalis* L*.*, Topical treatment

## Abstract

**Background:**

Medicinal plants have been used traditionally since centuries for wound care and treatment of skin diseases both in human and animals. Skin diseases are one of the most common reasons for owners to take their dog to the veterinarian. The demands for treatment and prophylaxis of these diseases are broad. A wide range of bacteria including antibiotic-resistant bacteria can be involved, making the treatment challenging and bear an anthropo-zoonotic potential. The aim of this review is to systematically evaluate based on recent scientific literature, the potential of four medicinal plants to enrich the therapeutic options in pyoderma, canine atopic dermatitis, otitis externa, wounds and dermatophytosis in dogs.

**Results:**

Based on four books and a survey among veterinarians specialized in phytotherapy, four medicinal plants were chosen as the subject of this systematic review: *Calendula officinalis* L. (Marigold)*, Hypericum perforatum* L. agg. (St. John’s Wort)*, Matricaria chamomilla* L. (syn. *Matricaria recutita* L., Chamomile) and *Salvia officinalis* L. (Sage). According to the PRISMA statement through literature research on two online databases a total of 8295 publications was screened and narrowed down to a final 138 publications for which full-text documents were analyzed for its content resulting in a total of 145 references (21 clinical, 24 in vivo and 100 in vitro references).

**Conclusions:**

All four plants were proven to have antibacterial and antifungal effects of a rather broad spectrum including antibiotic-resistant bacteria. This makes them an interesting new option for the treatment of pyoderma, otitis externa, infected wounds and dermatophytosis. Marigold, St. John’s Wort and Chamomile showed wound-healing properties and are thus promising candidates in line to fill the therapeutic gap in canine wound-healing agents. St. John’s Wort and Chamomile also showed anti-inflammatory and other beneficial effects on healthy skin. Due to the wide range of beneficial effects of these medicinal plants, they should be taken into account for the treatment of dermatologic diseases in dogs at least in future clinical research.

**Electronic supplementary material:**

The online version of this article (10.1186/s12917-019-1854-4) contains supplementary material, which is available to authorized users.

## Background

Skin diseases are one of the most common reasons for owners to take their dog to the veterinarian [1, 2]. Canine skin diseases include bacterial skin infections, hypersensitivity disorders, canine atopic dermatitis (CAD), food adverse reactions, otitis externa, wounds, dermatophytosis, neoplasia and parasitic infestations [2, 3]; a range of diseases with various etiologies and symptoms and thus diverse demands for treatment. An overview of these diseases with their possibly involved pathogens, pathogenesis and pathophysiology and demands for therapy and prophylaxis is presented in Table 1.

**Table 1:** Overview of the pathogens (and other often isolated microbes), pathophysiology, pathogenesis, clinical signs and the resulting demands for topical prophylaxis and therapy of five skin diseases in dogs

Disease complex	Opportunistic pathogens and other often isolated microbes		Pathophysiology/and pathogenesis/	Main clinical signs	Demands for topical prophylaxis and therapy
	Bacteria	Fungi			
Pyoderma (pyotraumatic dermatitis, bacterial overgrowth syndrome, intertrigo, furunculosis, mucocutaneous pyoderma etc.)^a^	*Staphylococcus pseudintermedius, Staphylococcus aureus, Pseudomonas aeruginosa,* Nocardia spp., Actinobacillus spp., Mycobacterium spp., *(Staphylococcus schleiferi),* Proteus spp., *Escherichia coli*		bacterial infection, (micro-)trauma, moist skin, erosion/ulceration, hemorrhage, inflammation	erythema, oedema), pain, alopecia, pruritus	astringent, wound healing, antipruritic, analgesic, antibacterial, anti-inflammatory
Canine atopic dermatitis^b^	*Staphylococcus pseudintermedius, Staphylococcus aureus*	*Malassezia pachydermatis*	allergens, skin barrier dysfunction, secondary infection, inflammation	erythema, pruritus	strengthening of skin barrier, antibacterial, antifungal, antipruritic, anti-inflammatory, anti-erythematous
Otitis externa^c^	Staphylococcus spp., Streptococcus spp., β-hemolytic Streptococci, Enterococcus spp., *Pseudomonas aeruginosa,* Corynebacterium spp. *(auriscanis), Acinetobacter baumannii, Proteus mirabilis*	*Malassezia pachydermatis, (Aspergillus fumigatus, Aspergillus niger, Aspergillus terreus)*	allergy, hypersensitivity, inflammation, secondary infection	pain	antibacterial, antifungal, anti-inflammatory, analgesic
Wounds (traumatic/bite wounds, surgical wounds, wound infections)^d^	Staphylococcus spp., Streptococcus spp., *Escherichia coli, Pasteurella multocida,* Neisseria spp., Corynebacterium spp., Moraxella spp., Enterococcus spp., Fusobacterium spp., Porphyromonas spp., Prevotella spp., Propionibacterium spp., Bacteroides spp., Peptostreptococcus spp., *Proteus mirabilis*		bacterial infection, hemorrhage, inflammation	pain	astringent, wound healing, antibacterial, anti-inflammatory, analgesic
Dermatophytosis^e^		*Microsporum canis, Trichophyton spp., (Microsporum gypseum)*	microtrauma	erythema, (pruritus)	antifungal, (anti-pruritic)

^a^[1, 128, 129]; ^b^ [4, 111, 130, 131]; ^c^ [7, 112, 132–136]; ^d^ [10, 11, 136, 137]; ^e^ [3]

Primary and secondary bacterial skin- and ear infections frequently occur in dogs. *Staphylococcus pseudintermedius* is the most common pathogen isolated from primary pyodermas as well as secondary skin and ear infections in dogs suffering from CAD or food allergies [4, 5] and carriage of methicillin-resistant *S. pseudintermedius* (MRSP) appears to be a risk factor for surgical site infections in dogs [6]. In addition, the opportunistic pathogen *Pseudomonas aeruginosa* is frequently isolated from ear infections and pyodermas in dogs as well [7].

Thus topical and oral antibiotics are frequently used in canine bacterial skin, ear and wound infections and the occurrence of resistance is a major concern. An increasing antimicrobial resistance in bacteria of the Staphylococcus intermedius group, isolated from clinical samples from dogs and cats has been reported [8]. Similar observations were reported for *P.aeruginosa* [7].

Treatment of infections associated with antimicrobial resistant bacteria can be very challenging. Moreover, the close contact between animals and their owners provides opportunities for microbial exchange, including MRSP or multi-drug resistant (MDR) pathogens [9]. It is therefore crucial to reduce the use of antibiotics and prevent further emergence of antimicrobial resistance and develop new antimicrobial therapies or develop measures that prevent microbial overgrowth. Microbial overgrowth is often associated with impaired epidermal barrier function such as seen in CAD. Therefore treatments that combine antimicrobial effects and anti-inflammatory or epidermal barrier enhancing effects would be even more desirable.

An additional emerging problem is the reported tolerance to the frequently used biocide chlorhexidine gluconate (CHG) solution which has been observed for some Gram-negative bacteria. Although mostly still effective against Gram-positive bacteria a recently confirmed link between antibiotic resistance and use of CHG demands for stewardship on the use of important biocides and thus there is a need for alternatives to the current biocides.

Another field affected by bacterial infections is wound care. The range of bacteria that can be found in infected wounds, especially bite wounds, is broad [10]. This makes exploring new treatment options like plant extracts interesting, even more so as medicinal plants consist of a mix of several compounds that may act against a broad spectrum of bacteria. However, not only infected wounds originating from injuries could profit from plant based antimicrobial agents but also certain surgical site infections. The latter can be associated with antimicrobial susceptible organisms but also MRSP or MDR pathogens [6, 11]. Also in the field of wound care there is a demand for new effective treatment options. Geriatrics and gerontology are of increasing importance in small animal care and even though the dogma that cutaneous wound healing is impaired as a function of age is not proven in dogs, structural and functional changes in the skin have been reported [12, 13].

Patients with impaired wound healing could benefit from substances supporting the healing process. Similarly, patients suffering from CAD could benefit from treatments which do not only fight bacterial and or yeast overgrowth/ infections but which also strengthen the skin barrier. While there are disinfectants and antibiotics registered for wound care, there are no registered veterinary drugs e.g. on the Swiss market (www.clinipharm.ch) which aid wound-healing itself.

Dermatophytosis in dogs occurs less frequently but is important because of its zoonotic potential [3]. Although current treatments are generally effective [3], additional treatment [14] options could be beneficial.

As numerous plant species have been used traditionally in Switzerland and other European countries for the treatment of various skin diseases including wounds [15–17], it might be an interesting option to look for alternative treatments in plant extracts. The goal of this review is thus to systematically evaluate the potential of four medicinal plants to enrich the therapeutic options in five relevant fields of canine dermatology: pyoderma, CAD, otitis externa, wounds and dermatophytosis.

## Methods

The methods of this systematic review are in accordance with the design of study by Ayrle et al. [18] and are based on the recommendations of the PRISMA statement [19, 20] and the AMSTAR measurement tool [21]. The PICOS scheme [19] was used to design the research question: the *population* are dogs, the *intervention* is a topical treatment with medicinal plants, the *comparator* is no treatment, a placebo or standard treatment, the *outcome* is the effect of the plant, the *study design* includes in vitro*,* ex vivo*,* in vivo or clinical trials. A detailed description of the systematic review is given in the Additional file 1.

### Selection of plant species

Four recent books on veterinary phytotherapy [15, 22–25] were manually screened by one person. Each plant mentioned as a remedy for dermatologic problems of any kind was listed and for each plant the number of books in which it was listed as such was counted. In order to also include the expertise and experience of specialists in the selection of plant species, a non-representative survey was conducted on veterinarians specialized in phytotherapy attending a conference in 2016. The 30 participants were given five minutes of time and were asked to spontaneously write down a maximum of ten plants they used most frequently as phytotherapeutic agents for dogs. They were also asked to give indications for their use. The received answers were filled into a table where each plant mentioned was listed together with the field of indication according to the ATCvet code [26]. A table describing the results of the screening of the books and the survey is accessible in Additional file 2.

The plants which were listed as therapeutic agent in dermatology in all four books or which were mentioned as such in three books and which were listed according to the ATCvet code D at least once in the survey, were included. The plants mentioned as dermatologically relevant therapeutic agents in all four books were *Matricaria chamomilla* L. (Chamomile) and *Calendula officinalis* L. (Marigold). Chamomile also has been mentioned by three contestants of the survey according to the ACTvet code D, and Marigold was mentioned four times. *Salvia officinalis* L. (Sage) was listed in three books and it was also mentioned according to the ATCvet code D three times in the survey. *Hypericum perforatum* L. agg. (St. John’s Wort) was listed in three books and was mentioned as a skin remedy by one contestant of the survey.

### Selection of scientific references

#### Literature search

For each of the previously selected plants an online bibliographic literature search was conducted. An introduction in scientific literature research was provided by a professional librarian. The databases used were PubMed [27] and Web of Science [28]. The literature search using these databases was conducted between 2017 and 05-03 and 2017-05-11 by one person. The search was limited on peer-reviewed publications published between 1997-01-01 and 2017-04-30. The search terms for the keyword search in both databases consisted of the Latin name of the plant, the English trivial names and the pharmaceutical denomination in Latin (e.g. *Salvia officinalis* OR Sage OR *salviae officinalis folium*). In the PubMed keyword search, the results were refined with the subjects “complementary medicine”, “systematic review”, “toxicology” and “veterinary science”. In the Web of Science keyword search the results were refined with the research areas “pharmacology pharmacy”, “integrative complementary medicine”, “toxicology”, “mycology”, “dermatology”, “veterinary sciences”, “infectious diseases”, “microbiology”, “virology”. In PubMed an additional MeSh-Term search was performed with the MeSh-Term of each plant (Latin name) and the subheadings “adverse effects”, “drug effects”, “microbiology”, “pharmacology”, “therapeutic use” and “toxicity”.

The use of plant extracts as anti-parasitic agents and in cancer therapy was not investigated as this would have exceeded the extent and the purpose of this review.

#### Keyword search within EndNote

For each plant species, a term-list search was conducted. Only references containing the word truncations “anti”, “astring”, “bioactiv”, “canin”, “constitu”, “derma”, “dog”, “eff”, “immune”, “pharma” or “wound” in their title or abstract were included. References containing “intest”, “gastro”, “pulmo”, “broncho”, “tumor” or “cancer” in their title or abstract were excluded.

#### Manual sorting of references according to in- and exclusion criteria

In order to further determine which publications were to be included in this review, the titles of the remaining publications were screened manually by one person and an in- or exclusion was made according to predefined in- and exclusion criteria. The references still included after the manual screening of the titles were screened and in- or excluded again by reading the abstract and applying the same criteria as stated above for the screening of titles.

#### In- and exclusion criteria

The in- and exclusion criteria were partially self-developed (based on Table 1) and partially derived from the publication by Ayrle et al. [18].

Only peer reviewed publications including an abstract written in English were considered. In order to be included, publications had to deal with an assessment of plants in vitro, ex vivo, in vivo or in a clinical trial and/or the topical use of plants and/or their extracts AND antimicrobial (antibacterial, antiviral or anti-mycotic) effects, anti-inflammatory effects, astringent effects, wound healing, epithelial proliferative or fibroproliferative effects, antipruritic effects, analgesic effects, other beneficial effects on the skin (e.g., anti-edematous, protective against water loss etc.), wound treatment, skin infections (bacterial, mycotic, viral), pruritus, otitis externa, seborrhea, CAD and other skin conditions occurring in dogs or being comparable to skin conditions occurring in dogs or dealing with disinfectant properties.

Publications dealing with ingredients, constituents, components of plants and detection or extraction of them were classified as “pharmacognostic publications”, and these were collected in a separate folder in EndNote for each plant. Publications referring to adverse effects or toxic effects of plants were classified as “publications on unwanted adverse effects” and were also collected in a separate folder in EndNote for each plant. Publications reviewing publications were also collected in a folder for each plant as “review publications”.

Publications without an abstract, which were only presented on conferences and not in peer-reviewed journals, investigating a mixture of different plant species in a combined preparation or dealing with other plant species than we focused on, were excluded. Also further publications were excluded: publications dealing with pathogens other than the main or closely related pathogens connected with dermatological disease in dogs (as shown in Table 1), plants used as food, plant genetics, cultivation or breeding of plants, plant pathology, plant protection systems or pesticides, ecology, geology, ethology, sociology, ethnobotany, food technology or food packaging, the use of the plant as a repellent, insecticide or anti-parasitic agent, homeopathic use of the plants (excluding mother tinctures, which were included), any anti-inflammatory or analgesic effects of a topically used plant that is not intended for skin problems (e.g. cream preparations for topical arthritis or migraine treatment), the effects of the plants and their extracts on endothelial cells, anti-inflammatory effects and effects on inflammatory cells but not explicitly connected to their role in the skin, effects of endophytic fungi and their products found in/on the plants, antiangiogenic properties if not connected directly to the skin or wound healing or the nutritional or mineral content of the plants.

#### Classification

Subsequently, the included (left over) publications were divided into the four groups “pharmacognostic publications”, “publications on adverse effects”, “review publications” and “finally included publications” (accessible in Additional file 3).

For the “finally included publications” the full text was acquired. One publication was excluded as the full text was not available.

Prior to filling the information of the “finally included publications” in a table (Additional file 3), a distinction between “finally included publication” and “reference” was made:

- **Finally included publication**: one scientific publication
- **Reference**: one or several clinical, in vivo or in vitro trial(s) of one plant species within one finally included publication (one line in Additional file 3). The references were classified into either “clinical”, “in vivo” or “in vitro” references. References investigating skin diseases occurring naturally in the investigated animal species (including human) were defined as “clinical references”. References investigating skin diseases and the effect of plants in human or animal models were categorized as “in vivo” references. Studies using pathogens, cell layers or ex vivo models were categorized as “in vitro” references. According to this definition, one finally included publication can contain several references (e.G. a publication on the same in vitro trial using several plants, e.G. Marigold, chamomile and sage, results in three references and one publication describing an in vitro and an in vivo trial with one plant species results in two references.) a publication reporting on several trials of the same category (clinical*,* in vivo*,* in vitro) results in one reference.

#### Scoring system

Based on the pre-defined demands for therapy and prophylaxis as described in Table 1, several possible effects of the plants and their extracts were added to the table (Additional file 3) as rows. If a reference showed one of these effects to be proven in a statistically significant way, it was marked as a “proven reference” and as a “+” in the table. If the reference was not able to show that an effect was proven or if the results were not statistically significant, it was marked as a “disproven reference” and as a “0” in the table. Uncertain effects presented in references (e.g. if the effect was not seen consistently) or if the reference did not state a clear result concerning that effect, these references were marked as an “uncertain reference” and a “?” in the table. If a reference showed the opposite of an effect (e.g. anti-angiogenic instead of pro-angiogenic) it would be marked as an “opposite reference” and a “-” in the table.

Ultimately, for each plant and each effect, the amounts of proven, disproven, uncertain and opposite references were counted and the results were presented.

## Results

**Fig. 1:**
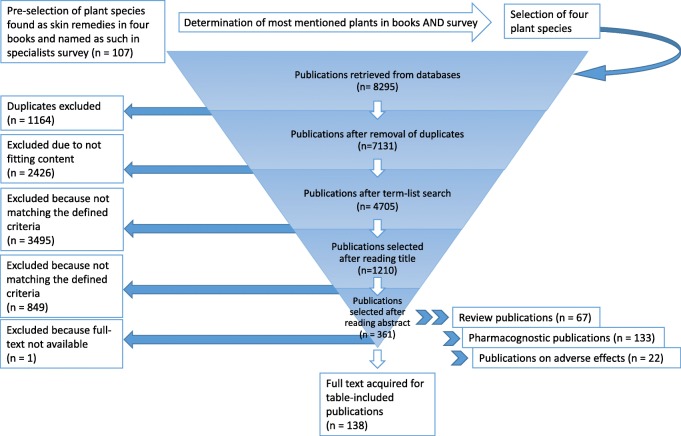
Process of the systematic literature review

Of the 8295 publications retrieved from databases, a final total of 138 publications were selected to be filled in the table, accessible in ‘Additional file 3’, resulting in a total of 145 references. The selection process of publications to be included into this table is described in Fig. 1 and a more detailed description of the number of publications found and included after different steps is provided in Table 2.

**Table 2:** Overview of the amount of scientific publications collected for each plant species after each step of the review process

Plant name (Latin)	*Calendula officinalis*	*Hypericum perforatum*	*Matricaria chamomilla*	*Salvia officinalis*
Plant name (English)	Marigold	St. John’s Wort	(German) Chamomile	Sage
All publications imported from WoS^a^ and PM^b^after removal of duplicates	536	2239	937	3319
After keyword search in titles and abstracts with EndNote	403	1728	683	1891
Included after manual screening of titles and abstracts	78	120	90	72
Pharmacognostic studies	36	42	21	34
Reviews	16	18	28	5
Publications on adverse effects	1	12	7	2
Finally included publications	25	48	34	31
Resulting references^c^	29	50	34	32

^a^Web of Science [28], ^b^PubMed [27], ^c^Reference = one or several clinical, in vivo or in vitro trials of one plant species within one finally included publication (= one line in dditional file 3)

**Fig. 2:**
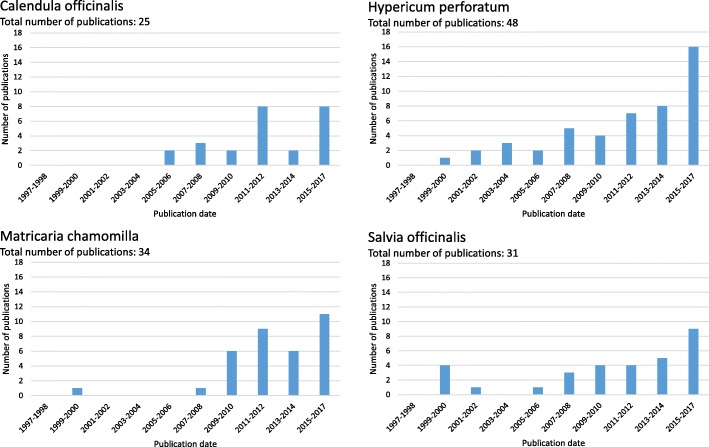
Distribution of the publication dates of all review included publications sub-divided by plant species

Figure 2 illustrates the distribution of the publication dates for each plant species over the 20 years included in this review in two-year-steps. For all plant species an increasing number of publications could be found for the last decade compared to the time period between 1997 and 2007.

**Fig. 3:**
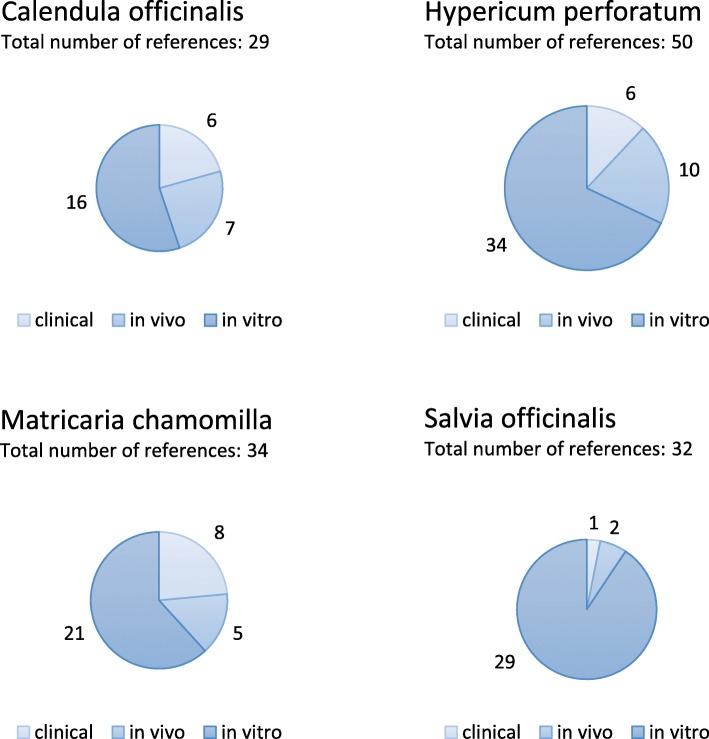
Distribution of the experimental design (in vitro, in vivo, clinical) of all review included references sub-divided by plant species

For each plant, the amount of total references resulting from the included publications and their distribution into clinical, in vivo and in vitro references is presented in Fig. 3. Most publications were included for St. John’s Wort and the most references resulted for this plant. Marigold was the plant with the least publications and references, but had the greatest percentage of in vivo and clinical trials. The highest percentage of in vitro references was found for Sage. Table 3 presents the number of in vitro and clinical/in vivo references which confirm (“+”), do not give a clear result for (“?”), do not confirm (“0”), or which show the opposite of (“-”) each of the total 16 effects required for the treatment of canine skin diseases. Overall, references showed seven times the opposite of the required effects while 198 times references were able to prove the required effect. Topical antibacterial, anti-fungal, anti-inflammatory and anti-erythematous effects were described in references of all four plants investigated. St. John’s Wort and Sage were the plants with the highest number of references investigating antibacterial effects and they were the only ones for which the activity against biofilms was studied and confirmed. Synergistic activities of plant extracts with antibiotics or disinfectants were only investigated for Chamomile and Sage. Sage was the only plant for which no studies related to wound-healing potential were found. For St. John’s Wort and Chamomile, publications investigating and showing analgesic effects were found.

**Table 3:** Summary of the amount of references proving (+), giving an unclear result (?), showing no effect (0) or showing the opposite effect (−) for each criterion investigated for each plant species

Type of reference		Calendula		Hypericum		Matricaria		Salvia	
		in vitro	in vivo + clinical	in vitro	in vivo + clinical	in vitro	in vivo + clinical	in vitro	in vivo + clinical
Anti-bacterial	+	7^1^		19^2^		11^3^	1^4^	20^5^	
	?			1^6^	1^7^				
	0			1^8^		3^9^		1^10^	
	–								
Synergism with antibiotics/ disinfectants	+					1^11^		4^12^	
	?								
	0							1^13^	
	–								
Anti-fungal	+	3^14^		10^15^		5^16^		7^17^	
	?								
	0	1^18^		4^19^		3^20^		3^21^	
	–								
Anti-biofilm	+			3^22^				2^23^	
	?								
	0								
	–								
Anti-pruritic	+				1^24^		3^25^		
	?						1^26^		
	0								
	–								
Wound-healing	+		8^27^		8^28^		5^29^		
	?				1^30^				
	0				1^31^				
	–								
Fibro-proliferative	+	3^32^	1^33^		1^34^				
	?			2^35^					
	0	1^36^			3^37^	2^38^	1^39^		
	–			2^40^	1^41^				
Fibro-migrative	+	2^42^		2^43^					
	?								
	0					1^44^			
	–			1^45^					
Collagen-enhancing	+	1^46^	2^47^	2^48^	4^49^		1^50^		
	?								
	0				1^51^				
	–								
Pro-angiogenic	+	2^52^	3^53^		3^54^				
	?								
	0				3^55^				
	–			1^56^					
Anti-inflammatory	+		1^57^	2^58^	3^59^		3^60^	1^61^	2^62^
	?		1^63^		1^64^				
	0				2^65^		1^66^		
	–	1^67^							
Anti-erythematous	+		1^68^		2^69^		2^70^		2^71^
	?						1^72^		
	0				2^73^				
	–								
Anti-edematous	+				3^74^		1^75^		1^76^
	?								
	0				1^77^				
	–								
Analgesic	+				2^78^		2^79^		
	?								
	0								
	–								
Beneficial for skin	+		2^80^		3^81^		1^82^		
	?								
	0				1^83^				
	–								
Hypothesis proven	+	2^84^	4^85^	5^86^	10^87^	2^88^	5^89^	3^90^	1^91^
	?						1^92^		
	0					1^93^	1^94^		
	–	1^95^							
Total	+	20	22	43	40	19	24	37	6
	?	0	1	3	3	0	3	0	0
	0	2	0	5	14	10	3	3	0
	–	2	0	4	1	0	0	0	0

^1^[62, 65–70]; ^2^ [53, 76–78, 138–152]; ^3^ [66, 68, 93, 97, 98, 100, 153–157]; ^4^ [158]; ^5^ [38, 93, 118, 119, 123–126, 157, 159–169]; ^6^ [170]; ^7^ [171];^8^ [172]; ^9^ [102, 103, 173]; ^10^ [174];; ^11^ [93]; ^12^ [93, 117–119]; ^13^ [167] ^14^ [65, 69, 175]; ^15^ [76, 79, 139–141, 151, 177, 178, 176, 179]; ^16^ [97]; ^17^ [120–122]; ^18^ [68]; ^19^ [78, 138, 152, 170]; ^20^ [68, 102, 103]; ^21^ [160, 166, 180]; ^22^ [79, 149, 150]; ^23^ [168, 181]; ^24^ [82]; ^25^ [35, 37, 104]; ^26^ [182]; ^27^ [29, 57–63]; ^28^ [31, 32, 82, 84, 87, 88, 183, 184]; ^29^ [104]; ^30^ [81];^31^ [185]; ^32^ [29, 64, 186]; ^33^ [29]; ^34^ [183]; ^35^ [45, 143];^36^ [187]; ^37^ [84, 86, 185]; ^38^ [64, 187]; ^39^ [105]; ^40^ [52, 64]; ^41^ [87]; ^42^ [29, 64]; ^43^ [45, 188]; ^44^ [64]; ^45^ [52]; ^46^ [189]; ^47^ [61, 62]; ^48^ [45, 188]; ^49^ [32, 86, 88, 183]; ^50^ [105]; ^51^ [185]; ^52^ [62, 190]; ^53^ [29, 62, 190]; ^54^ [86, 88, 183]; ^55^ [84, 87, 185]; ^56^ [191]; ^57^ [62]; ^58^ [33, 83]; ^59^ [33, 84, 85]; ^60^ [35, 104, 109]; ^61^ [192]; ^62^ [55, 193]; ^63^ [61]; ^64^ [88];^65^ [86, 87]; ^66^ [105]; ^67^ [189]; ^68^ [71] ^69^ [81, 194]; ^70^ [37, 109]; ^71^ [55, 193]; ^72^ [182];^73^ [88, 92]; ^74^ [32, 81, 82]; ^75^ [109]; ^76^ [55]; ^77^ [88]; ^78^ [81, 82]; ^79^ [104, 113]; ^80^ [71, 72] ^81^ [84, 86, 87]; ^82^ [115]; ^83^ [92] ^84^ [29, 50]; ^85^ [29, 30, 60, 195]; ^86^ [33, 52, 83, 143, 191]; ^87^ [31, 81, 82, 88, 92, 171, 185, 194]; ^88^ [34, 36]; ^89^ [35, 37, 108, 109, 115]; ^90^ [39, 120, 192]; ^91^ [38]; ^92^ [182];^93^ [196]; ^94^ [197]; ^95^ [189]

In the category “hypothesis proven”, various skin relevant effects were shown (Additional File 3). For Marigold, these effects included an increased expression of connective tissue growth factor and α-smooth muscle actin which was shown in an in vitro [29] as well as in an in vivo study [29], the increased expression of hyaluronic acid in human dermal fibroblasts and a protective effect against the induction of irritant contact dermatitis [30]. For St. John’s Wort, a protective effect on hair follicles and collagen was shown if applied directly after thermal burns [31]. Another study proved an inhibitory effect on hypodermal stasis in burn wounds indicating a protective effect on the perfusion in this area, preventing it from further damage through ischemia [32]. Two references reported an inhibitory effect of St. John’s Wort extract and hyperforin on the allostimulatory capacity of epidermal cells in vivo and in vitro, thus proving to decrease the epidermal cells’(EC) ability to present alloantigens in a mixed EC lymphocyte reaction and inhibit T cell proliferation [33]. Chamomile was proven to have a proliferative effect on keratinocytes in vitro [34] and in one study, the topical application of Chamomile essential oil lowered the serum IgG1, histamine and IgE level in atopic dermatitis-like mice [35]. It was also proven that Chamomile can increase the permeability of skin for caffeine and salicylic acid [36] and has good hair care properties in shampoo formulations [37]. Sage was shown to reduce axillary malodor levels when used in a deodorant stick [38] and an in vitro study showed that Sage ethanol extract inhibited NGF-induced neuritic outgrowth in an in vitro study based on a PC12 cell-line [39].

## Discussion

The large number of publications from the last twenty years shows a sound base of evidence-based knowledge about these four medicinal plants. Although the number of publications focusing on skin related topics is smaller, the amount is still in a promising range. There is a large number of pharmacognostic publications (a total of 133 for all four plant species) indicating that a broad knowledge on the constituents of the investigated plants is available.

The distribution of publication dates of the publications fitting the topic of this review indicates that there is a trend in investigating the therapeutic potential of these four plants in the context of skin diseases. Interestingly, the amount of publications found on St. John’s Wort (*H. perforatum*) is larger than for the other plants. St. John’s Wort has been receiving quite some attention in the last few years as many consumers are already using this medicinal plant for various indications such as depression therapy, urging researchers to investigate on the broad spectrum of effects of this plant [40].

### Evaluation of the search strategy

English or German scientific publications investigating the topical use of *Calendula officinalis, Hypericum perforatum, Matricaria chamomilla* and *Salvia officinalis* on dog skin are missing in the last 20 years. Due to this, the goal of this review was to extrapolate therapeutic options of the four medicinal plants for the topical use in dog skin based on studies investigating effects of these plants in other animals’ (and human) skin, microorganisms involved in canine skin diseases or closely related to those, cell cultures and other skin in vitro models. In order to avoid source selection bias, four standard textbooks and a survey among experts were used to choose the four most promising plants. It is, however, possible that the chosen books and the use described by the experts in the survey were influenced by each other which would result in a certain sampling bias. Using two different, independent databases and the Mesh Terms function of PubMed reduced the risk of introducing database bias. Also, by limiting the included publications to studies published not before 1997, some important but older research may have not been included in the present review. On the other hand, studies published up to 2017 were included, decreasing this bias.

### Varying chemistry of plants

Interpreting the results of studies investigating effects of plant extracts, it is important to consider that results may vary depending on the type of extract and the extraction method used [41, 42] and the amount of active constituents in the plant material. The amount of active constituents in the plant material itself can be influenced by several environmental factors [43]. Variation between the used plant parts and between subspecies of the plants needs to be considered as well [44, 45]. But the widely varying concentrations of active constituents in traditionally home-made plant-based remedies nevertheless seems to lead to a high level of satisfaction with the outcome of their use by Swiss farmers [46]. A problem is that not all available studies precisely describe the extracts used and therefore a minimum standard on how to report on the constituents should be requested for future studies [47]. To extract studies with comprehensible data and to create comparable units based e.g. on the concentration of raw material in the final preparations is possible but would go beyond the scope of this review.

### Multicomponent composition

The range of pharmacognostic publications found and considered for this review show that there is a broad spectrum of components found within one medicinal plant species, and most of the effects shown cannot be traced back to only one but multiple components of the plants and their extracts. For many effects, the groups of constituents causing them are known. For Chamomile for example, the components showing anti-bacterial activity were identified to be coumarins, flavonoids, phenolic acids, fatty acids and essential oil components [48]. The component bisabolol - which can also be found in Marigold [49] - is known to have anti-irritant and anti-inflammatory properties [37]. Oleanolic acid extracted from Marigold has been proven to be active against Gram-positive and Gram-negative bacteria [50]. A well-known component of St. John’s Wort is hypericin, a substance which is responsible for the photo-sensitizing effects of the plant [51]. Another ingredient found in St. John’s Wort, hyperforin, has been shown to have anti-proliferative and anti-migrative effects on fibroblasts [52] and α-pinene seems to be one of the components causing the antibacterial activity of St. John’s Wort [53]. Also for Sage, a wide range of components has been described. The terpene fraction of this medicinal plant is involved in many of the effects of Sage as it contains α- and β-thujone, 1,8-cineole, linalool, camphor and a range of other substances [54]. Ursolic acid from Sage has been shown to be one of the components responsible for its anti-inflammatory potential [55].

#### *Calendula officinalis* - Marigold

A well-known traditional indication in Switzerland for the use of Marigold is wound care [15, 56]. This field of use can be well justified by the results of this review as 8 clinical and in vivo references confirmed this effect [29, 57–63]. There were no references showing any result different than Marigold aiding the healing process. According to the publications assessed in this review, this effect can mainly be attributed to the fibro-proliferative, pro-angiogenic and collagen-enhancing effects of Marigold which were confirmed in several clinical, in vivo and in vitro references and also the fibro-migrative effect shown in two in vitro references [29, 64].

Seven references investigating the anti-bacterial effects of Marigold were identified, and all of them confirmed Marigold to inhibit the growth of skin-relevant bacteria [62, 65–70]. Even though the anti-bacterial activity of Marigold was not reported to protect against a broad spectrum of bacteria, and even though it may not be the strongest compared to other plant extracts [68], it still adds to Marigold being an interesting option for the treatment of wounds as it combines wound-healing and antibacterial effects.

Three in vitro references also confirmed an antifungal effect and only one in vitro study did not detect any anti-fungal activity of Marigold. This might justify the traditional use of this plant in fungal skin infections [15]. Two references confirmed beneficial effects on the skin such as an increase in skin moisture content, a decrease in the trans-epidermal water loss and an increased firmness of the skin [71, 72]. Such properties might also be interesting for the topical treatment of dogs with CAD as the barrier function of the skin is impaired in these diseases. Again, Marigold could possibly be a treatment fighting secondary infections and strengthening the skin at the same time.

Marigold has also been used traditionally to treat skin inflammation [15]. But the references included in this review show contradictory results and do not provide very strong evidence for the usefulness of Marigold in the treatment of canine skin inflammation [62, 63, 72, 73].

One publication on unwanted adverse effects was found describing contact sensitization to Marigold in humans affecting approximately 2% of the patients tested [74]. There are some other reports describing this problem to be more or less severe [73, 75] but none of the 13 in vivo and clinical references included in this review reported on any unwanted adverse effects. Also, one of these references conducted an irritant assay of a cream containing 1% of Marigold extract, demonstrating absence of skin irritation and a low irritability index in rabbits’ eyes [60]. Thus, marigold’s safety remains controversial.

Based on the strong evidence supporting the wound-healing effect of Marigold, this plant can be considered beneficial in canine wound care. As there are no veterinary drugs e.g. on the Swiss market which support wound healing other than just by disinfecting the wound, Marigold could fill a gap in therapeutic needs by aiding the healing process. The beneficial effects of Marigold on the skin could be used as a component of shampoos or sprays for normal skin care in healthy dogs as well as for dogs with skin problems.

#### *Hypericum perforatum* – St. John’s Wort

St. John’s Wort is a traditionally used remedy for skin alterations and sores in livestock [56], making it interesting to further investigations on effects and possible applications on dog skin.

The most frequently confirmed skin related effect of St. John’s Wort in this review was the antibacterial activity: a total of 21 references investigated this and 20 of them confirmed its antibacterial properties. It inhibited not only Gram-positive but also Gram-negative bacteria while generally showing stronger activity against the Gram-positive bacteria such as *S. aureus, S. epidermidis* and *E. faecalis*. *St. John’s Wort* was also proven to be effective against *E. coli, P. aeruginosa, Proteus mirabilis, Proteus vulgaris, S. epidermidis,* several *Streptococcus spp., Propionibacterium acnes,* several *Mycobacterium spp.,* and *Clostridium histiolyticum.*

Three references confirmed the activity of St. John’s Wort against methicillin-resistant *S. aureus* (MRSA) [76–78]. One reference also confirmed activity against gentamycin-methicillin-resistant *S. aureus* [76]. Thus, St. John’s Wort could be a new promising option for treatment of skin infections with Gram-positive bacteria including possibly MRSP. Another possible field of use for St. John’s Wort might be fungal skin infections as out of a total of 14 references investigating this (all of them in vitro), 10 did confirm the antifungal activity of the plant. Six references showed St. John’s Wort to be effective against dermatophytes including *Trichophyton mentagrophytes*, *Microsporum gypseum*, *Microsporum canis* and *Trichophyton rubrum*. There were no references investigating the effect of St. John’s Wort on *Malassezia pachydermatis* but one reference investigated and proved its activity against *Malassezia furfur* [79]. This shows that St. John’s Wort might inhibit the growth of a *Malassezia* species indicating that it might just as well be active against *M. pachydermatis*. Another factor in support of its anti-yeast activity is the fact that in eight references efficacy against *Candida albicans* was demonstrated. However *C. albicans* is infrequently causing infections in dogs but rather in humans but in general *Candida* spp. are even more resistant to azole antifungal agents than *M. pachydermatis* [80]. Therefore, it might be worthwhile to confirm efficacy of St John’s wort for *M. pachydermatis* as well. St. John’s Wort has been proven to be effective in 8 out of 10 references investigating its wound healing potential in in- and excisional wounds as well as experimental thermal burns in rats and in two human clinical trials using St. John’s Wort for the treatment of pressure sores and wounds after a cesarean section. The mechanism of the wound healing activity has not been identified as the references included in this review investigating fibroproliferative and fibromigrative effects as well as collagen-enhancing and pro-angiogenic properties reported rather mixed results. Still, the references evaluating the wound-healing potential of St. John’s Wort indicate that it can be helpful for wound care and as it has a rather broad-spectrum antimicrobial activity, it would qualify especially well for the treatment of infected wounds including bite wounds. Additionally, there were two references investigating and proving topical analgesic effects of St. John’s Wort [81, 82], which might present an additional reason to use this plant for wound care.

The results of references considering the anti-inflammatory effect of St. John’s Wort do show a tendency towards beneficial effects: while five references confirmed an anti-inflammatory effect [33, 83–85], two could not confirm it [86, 87], and one reference did not show a clear result [88]. Two references show significant anti-erythematous and three references edema reducing effects of St. John’s Wort (Table 3). Future studies need to be performed in order to investigate St. John’s Wort’s anti-inflammatory properties for the treatment of skin disease.

Three references showed further beneficial effects like an increased tensile strength (measured with a tensiometer) of the skin after external St. John’s Worts wound treatment, an interesting aspect maybe not only for the treatment of wounds. However, the cellular process behind this finding remains unclear.

Side effects of the use of St. John’s Wort preparations have been discussed controversial. Photosensitization was reported for the topical use of St. John’s Wort in some studies [51, 89, 90]. On the other hand a recent safety assessment excerted by the Cosmetic Ingredient Review Expert Panel stating that St. John’s Wort as used in cosmetic emulsions is rather safe [91]. One reference included in this short review tested a bath oil containing St. John’s Wort on human skin and found it to be less irritating than other tested bath oils and sodium lauryl sulfate. It was well tolerated having the same effects on the skin as the negative control, which was distilled water [92].

Considering all these different aspects St. John’s Wort may be helpful in the treatment of diseases like dermatophytosis, pyoderma/bacterial overgrowth, otitis externa and infected wounds. Even if clinical research with St. John’s Wort formulations in dogs is still missing it might be considered beneficial for topical applications in the treatment of canine skin diseases, maybe also as therapeutical shampoos.

#### *Matricaria chamomilla* - Chamomile

The traditional use of Chamomile for the treatment of skin-related problems in livestock seems to be well-established in Switzerland [17] and farmers using it appear to be content with its effects [46]. This indicates that there is a certain potential for Chamomile to be an effective treatment in canine skin disorders as well.

For Chamomile 14 in vitro references evaluating the antibacterial activity of its extracts were found and 11 of them could show an effect. The in vitro references could show inhibitory or even bactericidal effects of Chamomile against a relatively broad range of bacteria including *E. coli, P. mirabilis, P. vulgaris, E. faecalis, A. baumanni, Porphyromonas gingivalis, S. aureus, S. epidermidis* and other *Staphylococci.* The antimicrobial activity seemed to be stronger against Gram-positive bacteria and some references only provided evidence for an effect against Gram-positive but not against Gram-negative bacteria. Still, the inhibitory effect of Chamomile on bacteria seems evident and in some references it did present itself as being broad-ranged. One in vitro reference demonstrated Chamomile crude extract to be effective against methicillin-resistant *S. epidermidis* and a synergistic interaction between oxacillin and Chamomile was reported [93]. *S. epidermidis* is an important member of the canine skin microbiome [94] and is not uncommonly isolated from canine skin and ear infections [95, 96]. This shows that not only formulations containing Chamomile alone should be considered as new treatments but also formulations combining Chamomile with certain antibiotics may enhance their antimicrobial efficiency. Eight references described the antifungal properties of Chamomile and five of them showed it to be effective against *M. canis, M. gypseum, T. mentagrophytes, T. rubrum, T. tonsurans, Aspergillus niger, Aspergillus fumigatus, Candida albicans* and each two species of *Trichophyton* and *Aspergillus* genus [97–101]*.* Three references did not show an antifungal effect against *C. albicans* [68, 102, 103]. There were no clinical or in vivo references investigating the antifungal potential of Chamomile. The in vitro references described above demonstrated that extracts of this medicinal plant can be effective against dermatophytes and certain yeasts.

Chamomile was the plant for which the most references investigating anti-pruritic effects could be included. One clinical reference did not show a clear result concerning this effect but two clinical studies on humans [37, 104] and one in vivo reference using an AD mice model [35] did report Chamomile and its constituent bisabolol to relieve itching. Even though there were no publications found studying this effect in dogs, it seems likely that Chamomile might have a similar effect on dog skin as on human and mouse skin. Formulations containing Chamomile could thus have an additionally beneficial effect in the treatment of secondary skin infections in atopic dogs, in wound healing and other skin diseases associated with pruritus.

Five publications were included in this review studying the wound-healing effect of Chamomile and sustaining the traditional use of this plant for wound care [56]. Chamomile was reported to be effective in the treatment of experimental traumatic tongue ulcers, linear incisional wounds, cutaneous burn wounds, and peristomal skin lesions in human colostomy patients [104–108]. One reference showed a collagen-enhancing effect of Chamomile [105], and three references demonstrated the topical anti-inflammatory effects of Chamomile [35, 104, 109] which might also aid wound healing.

It is not surprising that besides the known and traditionally used systemic anti-inflammatory effect of Chamomile [15, 110], it also showed this effect when applied topically. Due to its anti-inflammatory effects it could replace the topical treatment with glucocorticoids not only in otitis externa but also in the management of CAD [111] eliminating the unwanted adverse effects resulting from the steroids [112]. Moreover, two references reported an analgesic effect of topically applied creams containing Chamomile in human patients [104, 113]. Even though there are reports of contact allergy to Chamomile in human patients [114] it is not known if dogs may show similar reactions. Considering the various beneficial effects of this medicinal plant it seems reasonable to investigate its use in dogs despite the unknown risk of provoking adverse reactions.

Due to its broad spectrum of promising scientific results Chamomile seems to be a therapeutic option in dogs suffering from otitis externa, pyotraumatic dermatitis and wounds. These patients might benefit not only from its anti-microbial and anti-inflammatory activity, but also from its analgesic effects.

One reference included in this review added Chamomile hydrophobic extract to dishwashing liquids in order to improve the safety of use. It was found that the extract reduced the transepidermal water loss and improved the skin hydration level in the human probands. Also, the more Chamomile extract was added to the formulations, the less irritant they were [115]. This effect should be considered in the development of dog shampoos for healthy skin and presents another beneficial effect of therapeutic shampoos containing Chamomile.

#### *Salvia officinalis* - sage

Sage leaf is used as a traditional relief of minor skin inflammations and bacterial infections of the skin in humans, and it has been known to have antibacterial properties [116]. The references found in this review showed that Sage is in fact effective against a range of bacteria relevant in skin disease. A total of 21 in vitro references investigated the antibacterial activity of Sage and 20 of them found an antibacterial effect. Fifteen references demonstrated that Sage inhibits the growth of *S. aureus*, seven references showed it to be effective against *E. coli* and six references found Sage to be effective against *P. aeruginosa*. Other bacteria against which Sage showed an inhibitory effect included *S. epidermidis* and other *Staphylococcus spp., Acinetobacter baumannii, Enterococcus faecalis, Enterococcus hirae, Propionibacterium acnes, Bacteroides vulgatus, Prevotella intermedia, Porphyromonas gingivalis, P. vulgaris, Proteus mirabilis* and other *Proteus spp.* and *several Streptococcus spp.* and *Corynebacterium spp*. In addition, methicillin-resistant *S. epidermidids* was inhibited by Sage as well as MRSA. These observations confirm that extracts of Sage exert broad-spectrum antibacterial activity and are effective even against resistant strains. There were no clinical or in vivo references included in this review which evaluated this effect of Sage on skin. Not only Sage extracts alone, but also combinations of them with antibiotics, seem to be very promising. A total of five references reported synergistic activities between antibiotics and one preservative and extracts of Sage and four of them could demonstrate such effects [93, 117–119]. Different extracts showed synergistic activity together with tetracycline, amoxicillin, chloramphenicol, oxacillin and the preservative propylparaben.

There were ten in vitro references investigating the antifungal activity of Sage. Out of these ten studies, seven reported it to be effective against a range of dermatophytes, yeasts and fungi including *T. mentagrophytes* [120–122], *T. rubrum, T. verrucosum* [120], *M. gypseum* [120–122], *M. canis* [120, 122], and *C. albicans* [120–126].

No reports on the potential adverse reactions to the topical application of Sage on dog skin were found. A reference evaluating the effects of different constituents of Sage on human gingival fibroblasts showed no toxicity. Thus, Sage can be considered relatively safe in its use on skin.

Given the promising results of the in vitro references proving the antibacterial effect of Sage, it would be interesting to further investigate the use of Sage in dogs with bacterial skin diseases, ear or wound infections. Sage was demonstrated to have anti-bacterial as well as anti-inflammatory properties. Combining these two properties, Sage seems promising for the treatment of primary or secondary skin infections and in the treatment of otitis externa. Also, the treatment of infected wounds could benefit from the anti-inflammatory component additional to the antibacterial effect. The references proving the antifungal effect of Sage showed that Sage could be effective in the treatment of dermatophytosis. Growth inhibition of *C. albicans* by Sage indicates a possible additional activity of Sage against *M. pachydermatis*, thus, indicating Sage’s possible properties in the treatment of skin and ear infections involving this yeast species [127]. This is especially of interest as treatment failure and rapid recurrences of these infections are common [127].

## Conclusions

All plants evaluated in this review possess a certain antibacterial potential and might be useful as a first-line topical treatment for primary and secondary skin infections, in surgical wound care, in wound infections and otitis externa. As antibiotic-resistant bacteria were shown to be susceptible to extracts of these plants, they present a possible new option for the treatment of dogs infected with germs resistant to antimicrobials. The same holds true for the antifungal effects of these four plants described. Hence, they might present an additional option for the treatment of dermatophytosis as well as skin and ear infections involving *M. pachydermatis* and even dermatophytes. Marigold, St. John’s Wort and Chamomile were reported to have wound-healing properties and thus, they represent promising candidates in line to fill the therapeutic gap in canine wound-healing agents. St. John’s Wort and Chamomile are the most promising species based on the findings of this review regarding anti-inflammatory effects. Thus, these plants may be used in a wide range of canine skin diseases with an inflammatory component. For the treatment of otitis externa and other painful skin conditions the topical treatment with St. John’s Wort and Chamomile should be considered.

Chamomile and St. John’s Wort have been shown to have beneficial effects even on healthy skin. These two plants could thus be an interesting additive for skin care products like dog shampoos or sprays. Ultimately, each of the plants investigated in this review combines several beneficial effects for the treatment of skin disease which makes them especially interesting due to their specific multicomponent composition. Some studies reported safety concerns in humans for these four medicinal plants. It is unknown if these apply to dogs too. However, as there are also several studies proving the use of these plants to be safe in humans, and as the beneficial effects of these plants are undeniable, these medicinal plants should be taken into account as therapeutic options for skin disorders associated with microbial infections/dysbalance and wound management in dogs, at least in future clinical research.

## Additional files

Additional file 1: Protocol of the systematic review. (DOCX 27 kb)

Additional file 2: 108 plant species connected to the treatment of skin disorders in recent books on veterinary phytotherapy and in a non-representative survey conducted on veterinarians specialized in phytotherapy. (XLSX 19 kb)

Additional file 3: Assessment of skin associated effects of four medicinal plants in 146 final peer-reviewed references. (XLSX 41 kb)
