# Supplementary material for: Medicinal plants as therapeutic options for topical treatment in canine dermatology? A systematic review
Source: BMC Vet Res. 2019 May 27;15:174. doi: 10.1186/s12917-019-1854-4 (PMC6537371; doi:10.1186/s12917-019-1854-4)
Supplement: Supplementary file 1 — Protocol of the systematic review. (DOCX 27 kb) [file 12917_2019_1854_MOESM1_ESM.docx]

Medicinal plants as a therapeutic option for the topical treatment in dog dermatology – A Systematic review

**Additional file 1: Protocol of the systematic review**

Aim of the systematic review:

The aim of this short systematic review is to assess if and in which fields of dog dermatology the extracts of the four medicinal plants *Calendula officinalis* L.*, Hypericum perforatum* L. agg.*, Matricaria chamomilla* L. and *Salvia officinalis* L. could be used as a treatment. The results of this review can present a base for experimental trials and clinical studies on dogs with skin problems.

Procedure:

1. Which are the most used plants in the treatment of skin disease including wounds?
   1. Screening of relevant initial sources based on secondary literature:

- 4 recent textbooks on veterinary phytotherapy

- 1. Conduction of a survey among specialists in phytotherapy asking:
- Which plants they use the most in their practice
- For which indications they use them
  1. Registering of all mentioned plant species from the books and the survey in a table
- Counting the amount of times every plant got mentioned in the survey according to the ATCvet code D
- Counting the amount of books each plant got mentioned in

1. Selection of four plant species

- Combining the counts of the table and choosing the four plants most mentioned in books and the survey

1. Searching for publications in online databases, based on an automatic search

3.1 PubMed

3.1.1 Search with keywords

- name or several names of the plant species in Latin and in English and the pharmaceutical name of the used part (e.g. “Calendula officinalis” OR “marigold” OR “ruddles” OR “Calendulae flos”)
- only publications published between 1997-01-01 and 2017-04-30, providing an abstract in English or German
- refining the results with “subjects”: complementary medicine, systematic review, toxicology, veterinary science
- saving the publications in an End-NoteX8-Data, for each plant in an own subfolder

3.1.2 Search with PubMed Mesh-Terms

- Mesh term of the plant (name of the plant species in Latin, e.g. Calendula officinalis)
- AND subheadings: adverse effects, drug effects, microbiology, pharmacology, therapeutic use and toxicity
- only publications published between 1997-01-01 and 2017-04-30, providing an abstract in English or German
- saving the publications in an End-NoteX8-Data, for each plant in an own subfolder

3.2 Web of Science

3.2.1 Search with keywords

- name or several names of the plant species in Latin and in English and the pharmaceutical name of the used part (e.g. “Calendula officinalis” OR “marigold” OR “ruddles” OR “Calendulae flos”)
- only publications published between 1997-01-01 and 2017-04-30, providing an abstract in English or German
- refining the results with “research areas”: pharmacology pharmacy, integrative complementary medicine, toxicology, mycology, dermatology, veterinary sciences, infectious diseases, microbiology, virology
- saving the publications in an End-NoteX8-Data, for each plant in an own subfolder

1. Removing the duplicates for each plant with the help of EndNote

The number of references for each plant is sampled and the used search terms and print screens of each search are stored.

1. Keyword search within EndNote

To refine the results, a term-list search within each plant species, including only publications which contain at least one of the predefined keywords in their title or abstract:

- Anti* (e.g. antibacterial, antifungal, antimicrobial, anti-inflammatory…)
- Astring*
- Bioactive*
- Canin*
- Constitu*
- Derma*
- Dog*
- Eff*
- Immune*
- Pharma*
- Wound*
- NOT: intest*
- NOT: gastro*
- NOT: pulmo*
- NOT: broncho*
- NOT: tumor*
- NOT: cancer*

The amount of remaining publications was sampled

1. Sample check of excluded references

To avoid the exclusion of relevant publications, the excluded ones are checked randomly by one person.

1. Relevance screening based on a manual procedure conducted through one person

Screening of the title (first step) and the abstract (second step) if the papers match the predefined inclusion and exclusion criteria. If the references do not fit those criteria, they are excluded. The amount of publications remaining after the first step and after the second step are sampled.

7.1 Inclusion criteria:
Inclusion of references which:

- Provide an abstract written in English
- Are published in peer-reviewed journals
- Investigate one of the four predefined plant, including different preparations like extracts or one single component of it

And dealing with:

a)

- An assessment of plants in vitro, ex vivo, in vivo or in clinical trials
- The topical use of plants/their extracts

AND

- Antimicrobial/antibacterial/antimycotic effects on skin skin relevant pathogen species
- Anti-inflammatory effects
- Astringent effects
- Wound healing or epithelioproliferative/fibroproliferative effects
- Anti-pruritic effects
- Analgesic effects
- Other effects on the skin (e.g, antiedematous, protective against waterloss etc.)
- Treatment of wounds, skin infections (bacterial, mycotic,), pruritus, otitis externa, seborrhea, atopic dermatitis and other skin conditions occurring in dogs or being comparable to the skin conditions occurring in dogs
- Disinfectant properties against skin relevant pathogen species

b)

- Ingredients, constituent, components of plants and the detection or extraction of them
- Toxic activity or side effects

7.2 Exclusion criteria
Exclusion of all references which:

- Do not provide an abstract
- Are only presented on conferences and not in peer-reviewed journals
- Investigate a mixture of different plant species in a combined preparation
- Dealing with other plant species or subspecies than the focused
- Do not mention a name clearly distinguishing the species or the latin name of the plant (e.g. only mentioning “chamomile” and not “German chamomile” or Matricaria chamomilla”)

Or dealing with:

- The effects of any other than the topical use of the plants
- Other animal classes than mammalians and birds
- Other medical branches of study, other diseases or apparatuses than mentioned in the inclusion criteria (e.g. gastroenterology, cardiology, oncology…)
- Other pathogens than the main pathogens or pathogens closely related to the main pathogens connected with dermatological disease in dogs
- Foodborne pathogens
- Plants used as food
- Plant genetics
- Cultivation or breeding with plants
- Plant pathology, plant protection systems or pesticides
- Ecology
- Geology
- Ethology
- Sociology
- Ethnobotany
- Food technology or food packaging
- The use of the plant as a repellent, insecticide or antiparasitic agent
- Homeopathic use of the plants (excluding mother-tinctures, papers on mother-tinctures are included)
- Any anti-inflammatory or analgesic effects of a topically used plant that is not intended for skin problems (e.g. cream preparations for topical arthritis or migraine treatment)
- The effects of the plants and their extracts on endothelial cells
- Anti-inflammatory effects and effects on inflammatory cells but not explicitly connected to their role in the skin
- Effects of endophytic fungi and their products found in/on the plants
- Antiangiogenic properties if not connected directly to the skin or wound healing
- The nutritional or mineral content of the plants

1. Data processing

8.1. Classification of the references regarding

a) different therapeutic needs based on pathophysiology and etiology of a range of skin diseases and

b) the investigated effects of plant preparations

Classification of therapeutic needs: antibacterial, synergistic activity with antibiotics/disinfectants, antifungal, anti-biofilm, anti-pruritic, wound-healing, fibro-proliferative, fibro-migrative, collagen-enhancing, pro-angiogenic, anti-inflammatory, anti-erythematous, anti-edematous, analgesic, beneficial for skin and “hypothesis proven” for effects on skin-relevant parameters being shown in less than three references.

8.2. Classification of the final remaining references in predefined categories referring with the type of study:

- *in vitro* studies = all studies conducted on cellular level and/or on pathogens and ex vivo studies
- *in vivo* studies = all studies using an animal model, which normally is not suffering from the disease / health problem
- clinical trials = all studies using an animal species (including human), which may suffer from the disease / health problem
- reviews = assessment and comparison of available literature on one topic
- pharmacognostic studies = all studies aiming the biochemical composition or the analysis of their components
- papers on adverse effects = studies investing the toxicity and adverse effects of the plant
